# The German diabetes self-help and its view to personalised medicine- the development of diabetes self-help in Germany since 2011

**DOI:** 10.1186/1878-5085-5-S1-A75

**Published:** 2014-02-11

**Authors:** Deutsche Diabetes-Hilfe – Menschen mit Diabetes

**Affiliations:** 1 Landesverband NRW e.V., Johanniterstraße 45, 47053 Duisburg, Germany

## 

The Diabetes Self-Help of the state North Rhine-Westfalia (NRW) exists since 1975 as an independent association. Till 2012 it was member in the German Diabetes Federation called Deutscher Diabetiker Bund (DDB).

On 8^th^ June 2012 in Berlin there was founded a new patient organisation called Deutsche Diabetes-Hilfe – Menschen mit Diabetes (German Diabetes Help – People with Diabetes) with the abbreviation DDH-M. This organisation works in the umbrella organisation diabetesDE – Deutsche Diabetes-Hilfe on the same level with the Association of Diabetes Specialists (Deutsche Diabetes Gesellschaft (DDG)) and the Association of the Diabetes Counselling Professions (Verband der Diabetesberatungs- und –schulungsberufe (VDBD)). The Diabetes Self-Help Organizations of NRW and three other German states became members of DDH-M.

Reasons for this change from DDB to the new organisation DDH-M were a better and closer cooperation with other umbrella organizations, specialist associations, scientific and social institutions as well as politicians and the possibility of networking with these team players.

## Measures considering prevention and Personalised Medicine

Although there is evidence that not all groups of patients benefit from all measures of Predictive, Preventive and Personalised Medicine (PPPM) we aim at guaranteeing access to PPPM for those groups of patients that definitely benefit from particular measures. It is our conviction that Personalised Medicine also has an economic benefit for our health system.

Since 2003 the Diabetes Self-Help NRW has a Diabetes-Info-Mobile. It is a car which is driven by a diabetes counsellor to do all kinds of prevention. By using an instant method for diagnosis with the equipment in our Diabetes-Info-Mobile, the counsellor gives advice and makes tests of the level of blood sugar, blood pressure and fat metabolism.

For the first time in November 2012 the Diabetes Self-Help NRW had two sessions with lectures at the autumn congress of Deutsche Diabetes Gesellschaft. Here prevention also was an important subject with the focus not only on medical aspects but also on economic and social issues.

Since March 2013 the Diabetes Self-Help NRW participates in the political prevention campaign “Stop Diabetes – Now!” along with DDH-M and the umbrella organisation diabetesDE.

## Keeping in contact with politicians

A lot of general conditions which are necessary to make use of preventive measures for people’s healthcare have not been initiated by politicians yet. It has always been the initiative of the Diabetes Self-Help Organisation to start projects with a preventive impact or screenings by means of the Diabetes-Info-Mobile. All these projects took place without any political support. This year, however, for the first time we had a successful feedback by talking with politicians.

The Diabetes Self-Help NRW talked with politicians from the NRW Ministry of Health and the health committee of the German government about prevention and Personalised Medicine. All politicians said that prevention is very important, but we noticed that they use a different definition of Personalised Medicine. Sometimes they reduce the meaning of Personalised Medicine to the level of genomic tests. But all of them assured that they are interested in Personalised Medicine. However there is no politician who can participate in the EPMA Congress because of the German governmental elections on 22^nd^ September.

## Cooperation of all team players

On suggestion of DDH-M Diabetes Self-Help NRW there was founded a new networking institution which is called Diabetes Initiative NRW. Participants are different representatives of specialist associations, organizations, institutions (medical professionals, therapists, counsellors, scientists) as well as insurance companies and other institutions which give financial input to our health system. Last but not least there are also some politicians and producers of medical equipment who are part of this initiative. It is the target of this networking group is to launch a draft of measures against the deficits in diabetes healthcare. The initiative has regular meetings.

## Information

To publish information and news of diabetes self-help we use different media: internet, the magazine “DDH-M aktuell” for members of our organisation as well as other diabetes magazines and newspapers if possible.

## Outlook on the next 2 – 3 years

We plan to extend counselling and prevention by our Diabetes-Info-Mobile.

Together with the umbrella organisation diabetesDE the Diabetes Self-Help NRW plans a project “Diabetes Counselling on Wheels” in cooperation with diabetes counsellors, diabetes specialists, pharmacists and urban health institutions. A part of this project is a prevention screening in regions with a high number of people with migratory background. At the end of August 2013 the German Ministry of HealthCare offered support of such a project if this project will be evaluated scientifically.

Together with the German Diabetes Centre (Deutsches Diabetes Zentrum) in Düsseldorf and a health insurance company we plan another project that is concerned with “Diabetes at School”. It is the target to sensitize teachers to the issue of children with diabetes.

The Diabetes Self-Help NRW and Kirchheim-Verlag, the publishing company of our magazine “DDH-M aktuell” and other diabetes magazines, also plan an event in cooperation with diabetes specialists, counsellors, the organisations of pharmacists, networks of foot diseases specialists and scientific institutions. We will also take part in other events, regional ones and supra-regional ones, for example at the congress of Deutsche Diabetes Gesellschaft. If possible we will then try to talk about prevention and personalised medicine.

Last but not least we hope that we can continue participating in activities of European Association for Predictive, Preventive and Personalised Medicine (EPMA). In the future there will not only be medical but also a high number of social and economic challenges in our health system. Therefore we need cooperation and networking of all European team players.

